# The Waste of Life from Cancer*An address delivered at La Grange, Texas, December 22, 1914, before the County Teachers’ Institute, under the auspices of the Lecture Bureau of the American Medical Association.

**Published:** 1915-07

**Authors:** John T. Moore

**Affiliations:** Fellow American College of Surgeons, Houston, Texas


### EDITORIAL DEPARTMENT.

MRS. F. E. DANIEL, Publisher and Managing Editor.
DR. R. H. L. BIBB, Associate Editor.

CONTRIBUTING EDITORS:

Dr. H. Sheridan Baketel, New York, editor *Medical Times;* Dr. M. H. Boerner' Austin, Texas; Dr. G. Henri Bogart, Paris, Ill.; Dr. M. M. Carrick, Dallas, Texas; Dr. Edward H. Carey, Dean Medical Department, Baylor University, Dallas, Texas; Dr. W. L. Crosthwaite, Waco, Texas; Dr. T. D. Crothers, Hartford, Conn.; Dr. H. W. Cummings, Hearne, Texas; Dr. Oscar Dowling, New Orleans, President Louisiana State Board of Health; Havelock Ellis, M. D., London, Eng.; Dr. May Agnes Hopkins, Dallas, Texas; Dr. A. W. Fly, Galveston, Texas; Dr. G. B. Foscue, Waco, Texas; Dr. E. S. Goodhue, Holualoa, Kona, Hawaii; Dr. M. B. Grace, Seguin, Texas; Dr. Joe Gilbert. Austin, Texas; Dr. Charles H. Hughes, St. Louis, editor *Alienist and Neurologist*; Dr. James G. Kiernan, Chicago; Dr. George H. Lee, Galveston, Texas; Dr. John T. Moore, Houston, Texas; Dr. Frank Paschai, San Antonio, Texas; Dr. John Preston, Austin, Texas, Superintendent State Lunatic Asylum; Dr. G. Wilse Robinson, Kansas City, Mo.; Dr. Bacon Saunders, Fort Worth, Texas; Dr. Allen J. Smith, Philadelphia, Medical Department, University of Pennsylvania; Dr. Z. T. Scott, Austin, Texas; Dr. Ralph Steiner, Austin, Texas, President Texas State Board of Health; Dr. Walter S. Sutton, Kansas City, Mo.; Dr. A. E. Sweatland, Nacogdoches, Texas; Dr. John S. Turner, Dallas, Texas; Dr. Charles Venable, San Antonio, Texas.

---

### The Waste of Life From Cancer.*

BY JOHN T. MOORE, A. M., M. D., FELLOW AMERICAN COLLEGE OF SURGEONS, HOUSTON, TEXAS.

The American people are frequently represented by foreign caricaturists, as wearing garments figured with the dollar sign. We cannot help excusing them somewhat, when it is considered how our judgments are formed of the success of human activities by the dollars and cents that are the result. The virtues of a life are too often lost sight of in our effort to place a material value upon them.

We all find ourselves asking, What is he worth in money and property? instead of seeing his great value as a citizen. We seem to be unable to estimate the value of our citizenship on any other basis.

We boast of our great material resources in the way of lands, mines, buildings, and other physical properties, which are estimated to be one hundred and eighty-seven billion dollars ($187,-000,000,000).

Now according to the studies of Prof. Irving Fisher ("National Vitality—Its Waste and Conservation") our vital assets are two and one-third times the value of our whole physical wealth.

We Texans are fond of holding up to the astonished residents

---

*An address delivered at La Grange, Texas, December 22, 1914, before the County Teachers' Institute, under the auspices of the Lecture Bureau of the American Medical Association.

of other States and countries our State's wide extent of boundary and her yet unmeasured resources in agriculture, stock raising and other physical resources; yet, in spite of this, we appreciate but poorly the greatest resource of wealth in our great State is our people themselves. The people are worth three to five times as much as the physical properties that we talk about so much.

We must study our stock in trade—vital and physical—and handle it in such a way as to produce the greatest good.

The laws of *efficiency* and *conservation* must be studied and applied all along the line.

All wealth is but the evidence of human energy and activity. The community, the State, or the nation is the richer according to its *efficiency,* and this is determined by the *conservation* of the human energy within their confines.

Every life being the greatest factor in the production of wealth. it is necessary that we plan to keep the people in good health and extend their period of useful activity as much as possible.

The world stands amazed at the advances made in medicine in the discovery of the cause, prevention and treatment of disease.

It is said, "The science of disease prevention, if properly applied, can add fifteen years to the average length of human life." At the present death rate of preventable diseases, over six million lives will be needlessly destroyed during the next ten years. Every fifteen seconds a life is lost from a preventable disease (*American Life Waste,* 1910).

Not so many years ago tuberculosis was a poorly understood disease, and very little was attempted to be done either to prevent it or to cure those sick of it. When one became affected, he was regarded as hopelessly sick, and left to ignorantly spread the infection, and to get well by accident. Now, we preach a very different gospel. We know how to prevent its spread, and cure many cases by carrying out the well known laws of cure—rest, good food, good air and sunshine. The disease grows less frequent as the laws of prevention and cure are carried into operation.

Cancer, the problem before us this evening for discussion, comes probably second in importance to that of tuberculosis.

It is being carefully studied in a similar way, and the same more hopeful view is being taken of its prevention and cure.

Cancer does not kill so many as does tuberculosis, yet it claims its victim, so often, just at the time of life when the man or woman is most valuable to the family and to the community.

Of the diseases that occur at and above 40, it is the most frequent and most dreadful. At the ages above 45, it is said that one-sixteenth of all deaths are due to cancer. The rate increases rapidly after this age. Of the women who live to the age of 40, one-eighth die of cancer, while one man in fourteen is taken off by this disease.

The total deaths from this disease equal 75,000 annually. This is startling enough, yet statistics seem to show that cancer is on the increase in all civilized countries.

The disease is said to prove fatal in about 90 per cent of the attacks, when, if the medical profession and the people could only be made to understand as much about it as they do of tuberculosis, there would be 75 to 90 per cent of cures, to say nothing of the many cases that would be prevented.

The desire on the part of the American Medical Association to contribute its part towards the spread of information about this disease and other diseases led them to the organization of a Lecture Bureau, under whose auspices I come to you, hoping to be able in some way to render a service that may to some extent prevent and lead to the cure of those afflicted with this terrible malady.

There are many of the leading medical men in each State giving freely of their time in a similar direction.

The people themselves are manifesting a great interest towards informing themselves upon these great questions of disease prevention and the cure of maladies that affect the whole human race.

I shall attempt to tell you in language simple enough to be understood by laymen what is known of this frightful disease.

I presume there are few present who cannot recall one or more cases of cancer that have come under your observation. You no doubt remember the various views and opinions expressed about the nature and the curability of the disease.

There may be an impression prevailing among you that there is little known about the cause of cancer. This impression is probably true, if we refer to the real specific cause of cancer—as we know the cause of tuberculosis—but, if we be allowed to speak of the conditions which produce the disease, we are able to tell you much.

In the past few years, much effort is being put forth by individuals, societies and medical institutions throughout the civilized world to discover, if possible, the cause of cancer. Much statistical information relating to the distribution of the disease, the effect of climate, habits of life of the different races, etc., has been collected.

Experiments of various kinds on the lower animals, in transplanting tumors, watching their growth, and studying the modifying influences of different conditions, have been carried out.

In our own country we have the American Society for the Control of Cancer doing much to spread information to the people concerning the nature of the disease.

This, as well as other organization, appreciates that, pending the discovery of the specific cause, and possibly a certain cure for cancer, much can be accomplished by working at its *prevention* and *control.*

Here we are to work largely in two directions, viz.:

1. With the medical profession.

2. With the people themselves.

1. The medical profession needs to study the disease more carefully and become familiar with all that is known about it. It is desirable that the profession learn all about the predisposing causes of cancer, so that they may teach their patients how to live in the safest way.

We now know from a very large and varied source of information that many lesions that are simple in themselves become cancerous. Bloodgood says *that cancer never begins in a healthy spot.* This statement is certainly true, hence the importance of us as physicians making a closer study of these defects upon which cancer so often grows.

There seem to be relatively few physicians who teach the importance of the removal of warts and small skin tumors, yet how often do we see these become cancerous?

Ulcers of the stomach, and obscure conditions of this organ, are left too long many times before resorting to curative measures.

There are still physicians who attach very little importance to abnormal bleeding in women. The lesions that are curable must not be allowed to become cancerous.

The medical profession must inform itself, thoroughly, of the best methods of treating all types and stages of cancer.

Many cancers that are curable are made worse and rendered incurable by improper methods of treatment.

The medical profession must know all of these things, hence medical schools, clinics and medical societies must help in every way possible to spread the information among doctors.

A careful study of the improved and radical methods of treating cancer has given us a more cheerful outlook for the cure of cancer.

This leads us to say that the doctor must preach the gospel of greater promise to the people. As we inform ourselves, we increase the percentage of cures. We must really come to believe and know the results are better before we are willing to preach a more hopeful gospel.

2. *The people themselves* must be informed regarding the nature of the disease and its curability, if properly treated in its early stages.

There is much discussion pro and. con about the desirability of attempting to teach the people about this disease. Some contend that the constant fear of cancer would do much harm. While same harm might result in this way, much more good would be accomplished by people having a sufficient amount of fear to lead them to consult and be guided by properly informed medical men.

So much of the information in the hands of the people is either worthless or harmful.

Now something about cancer: The term "cancer" is generally used in reference to any malignant growth; that is, those growths that tend to spread in their growth in such a way as to take the life of the individual.

Simple growths in contradistinction to the malignant growths stay in one place, and do not spread to other tissues. These simple growths sometimes kill by pressure, or injury to other organs, while malignant or cancerous tumors are taken either through the blood or lymphatic channels, and there implanted, forming new tumors. These may spread to many different parts of the body.

The body, as you all now know, since most of us have studied enough anatomy and physiology to get this information, is made up of organs, and these of a certain kind of small cells. These arrangements of cells make up certain kinds of tissues.

Now these tissues grow in certain definite ways; i. e., their cells do. The body, with its different tissues and organs, reaches its development or size and remains about the same, carrying on its varied functions.

For some reason, caused by something whose exact nature we do not know, the cells at a certain point begin to grow, and these continue in this wild uncontrolled fashion until, if left alone, are apt to take the life of the individual.

It is now pretty well known that heredity plays no direct part in producing cancer. There may be inherited a slight predisposition to the disease, though many of our best observers deny this.

The evidence collected in recent years seems to show that cancer is not contagious.

One need have no fear of contracting the disease by contact with people who have it.

The laity should remember that cancer always grows upon some unhealthy spot. There is practically always a history of a lesion that antedated the cancer. These abnormalities should always have attention by competent medical advice.

I desire to conclude with the quotation of the statement made by the Borough of Portsmouth; e. g., in relation to cancer:

"The only cure for cancer, at present known, is its early and complete removal. Cancer, if removed early, has been proved conclusively to be a curable disease. If neglected, and not removed in its earliest stages, it is practically invariably fatal. The paramount importance of its early recognition and early removal is therefore evident. For this purpose, the assistance both of the public and the medical profession is requisite, and a grave responsibility rests on both. It is only by their mutual co-operation that the ravages of this terrible disease can be lessened.

"The following information should be of vital assistance to the public. It is no exaggeration to say that, if acted upon, the result would be the saving annually of many hundreds of lives which at present are inevitably lost:

"1. Cancer, in its early and curable stages, gives rise to no pain or symptoms of ill health whatever.

"2. Nevertheless, in its commonest situations, the signs of it in its early stage are conspicuously manifest, to witness:

"3. In cases of swelling occurring in the breast of a woman after 40 years of age, a medical man should at once be consulted. A large proportion of such swellings are cancer.

"4. Any bleeding, however trivial, occurring after the change of life, means almost invariably cancer, and cancer which is then curable. If neglected till pain occurs, it means cancer which is almost always incurable.

"5. Any irregular bleeding occurring at the change of life should invariably be submitted to a doctor's investigation. It is not the natural method of the onset of the change of life, and in a large number of cases means commencing cancer.

"6. Any wart or sore occurring on the lower lip in a man after 45 years of age is almost certainly cancer. If removed at once the cure is certain; if neglected the result is inevitably fatal.

"7. Any sore or swelling occurring on the tongue or inside of the mouth in a man after 45 years of age should be submitted to investigation without a moment's delay, and the decision at once arrived at by an expert microscopical examination whether it is cancer or not. A very large proportion of such sores or swellings occurring at this time of life are cancer, and if neglected for even a few weeks the result is almost inevitably fatal. If removed at once the prospect of cure is good.

"8. Any bleeding occurring from the bowel after 45 years of age, commonly supposed by the public to be "piles," should be submitted to investigation at once. A large proportion of such cases are cancer, which at this stage is perfectly curable.

"9. When warts, moles or other growths on the skin are exposed to constant irritation, they should be immediately removed. A large number of them, if neglected, terminate in cancer.

"10. Avoid irritation of the tongue and cheeks by broken, jagged teeth, and the lower lip by clay pipes. Many of these irritations, if neglected, terminate in cancer.

"11. It is desirable that rooms occupied by a person suffering from cancer should be cleaned and disinfected from time to time."

While perhaps some of the statements in this circular may possibly be open to discussion and amendment, as a whole, it admirably states the principal facts in regard to the disease which should be known to every adult person. Similar circulars have already been published by the American Society for the Control of Cancer, and it is just such facts that the Society will repeat on every possible occasion until they become as commonplace as our present knowledge of the main facts in regard to tuberculosis.

